# Ultrasound Brain–Computer Interfaces for Transcranial Closed-Loop Neuromodulation

**DOI:** 10.34133/bmef.0312

**Published:** 2026-09-17

**Authors:** Chuhao Yin, Juan Tu

**Affiliations:** ^1^ Department of Acoustics, School of Physics, Jiangsu Key Laboratory for Cardiovascular Information and Health Engineering Medicine, Nanjing University, Nanjing 210093, China.; ^2^ School of Integrated Circuits, Nanjing University of Information Science and Technology, Nanjing 210044, China.

## Abstract

Noninvasive brain–computer interfaces (BCIs) have advanced communication, assistive control, and neurorehabilitation, but most remain readout-oriented systems with limited capacity for deep targeting, focal intervention, and response-guided modulation. Ultrasound offers a distinct route to brain interfacing: functional ultrasound imaging provides spatially localized hemodynamic readout, although current human task-related evidence relies on surgically enabled acoustic access; transcranial focused ultrasound enables noninvasive modulation of cortical and subcortical circuits. We define an ultrasound BCI as a system that converts measured brain activity into a functionally useful output, with ultrasound providing readout, state-contingent write-in, or both; in the closed-loop framework considered here, response assessment updates or constrains subsequent modulation. This Perspective argues that ultrasound BCI should be viewed not as a replacement for established BCIs, but as a transcranial closed-loop acoustic neuromodulation platform. We clarify its boundaries, compare current evidence maturity, and outline translational routes for neurorehabilitation, abnormal brain-state intervention, and deep-brain neurofeedback.

## Background: From Noninvasive Readout to Transcranial Closed-Loop Acoustic Neuromodulation

Noninvasive brain–computer interfaces (BCIs) have progressed from proof-of-concept demonstrations toward translational use in communication, assistive control, and neurorehabilitation [1]. Following the BCI Society working definition, we treat the near-real-time conversion of measured brain activity into functionally useful outputs that change ongoing brain–environment interactions as the defining requirement of a BCI; targeted stimulation may additionally provide functionally useful inputs to the brain [2]. Electroencephalography (EEG) remains the dominant online readout modality because of its millisecond-scale temporal resolution, mature decoding pipelines, and relatively low deployment barriers. Functional near-infrared spectroscopy (fNIRS) complements EEG by enabling wearable hemodynamic sensing, whereas magnetoencephalography (MEG) and emerging optically pumped magnetometer MEG systems are extending high-fidelity noninvasive recording toward less constrained behavioral settings [3]. These advances have positioned noninvasive BCI as a translational neurotechnology, but also underscore that most current systems remain primarily readout-oriented.

This readout-centered architecture becomes limiting when the clinical goal requires intervention. Conventional noninvasive BCIs can infer user intention, task state, or changes in neural activity, but they rarely combine deep-target access, focal neuromodulation, dose traceability, and response monitoring within the same platform. For therapeutic applications, a key question is therefore not only how to read brain states faster and more accurately, but also how to link decoded states to targeted, adjustable, and verifiable neuromodulation. This question is especially relevant to poststroke motor rehabilitation, intervention for abnormal brain states, consciousness assessment, and deep neurofeedback, where the clinical objective is not simply external command output, but sustained coupling among brain state, feedback training, intervention dose, and functional recovery.

Ultrasound-based neural technologies have provided foundational capabilities on both the readout and intervention sides [4]. Functional ultrasound (fUS) uses ultrafast Doppler imaging to detect changes in cerebral blood volume or blood flow [5] and has since been extended to task-related brain imaging in behaving nonhuman primates [6] and single-trial decoding of movement intention [7]. Current human task-related fUS demonstrations with spatially resolved activity mapping have relied on surgically placed sonolucent cranial implants [8,9]. By contrast, intact-skull human fUS remains at the proof-of-concept stage and has not yet demonstrated task-related neural decoding or online BCI operation [10]. Transcranial focused ultrasound (tFUS) can modulate cortical and subcortical neural activity through noninvasive, spatially focused acoustic energy delivery, with evidence ranging from human cortical modulation [11] and primate deep-brain modulation [12] to BCI performance enhancement [13] and a system for human deep-brain neuromodulation [14]. Ultrasound is therefore entering the BCI field not because readout or stimulation tools are lacking, but because it offers a route to combine localization, functional readout, acoustic write-in, and response feedback within a unified transcranial acoustic framework.

Accordingly, in this Perspective, an ultrasound BCI computationally converts brain activity measured by fUS or another neural modality into a functionally useful output, with ultrasound providing fUS readout, state-contingent tFUS write-in, or both. Closed-loop systems additionally use measured responses to update or constrain subsequent actions, establishing a traceable state–action–response relationship. Acoustic localization or dose planning alone, passive fUS imaging without a functionally useful output, and preset open-loop tFUS do not, by themselves, constitute an ultrasound BCI.

## What Makes Ultrasound BCI Different from Conventional Modalities

Ultrasound differs from conventional BCI modalities through its physical field pathway. EEG, MEG and fNIRS provide low-risk noninvasive readout, but they do not inherently provide focal write-in through the same physical channel; implantable BCIs can provide high-resolution recording and stimulation, but at the cost of invasiveness and long-term stability constraints. Ultrasound occupies a distinct intermediate position: acoustic fields can be transmitted through tissue, focused across the skull and parameterized for both sensing and intervention. However, transcranial propagation is constrained by skull attenuation, aberration, and interindividual variability [4,15]. This acoustic-field controllability provides a physical basis for linking fUS-based hemodynamic readout with tFUS-based acoustic neuromodulation. Representative condition-specific trade-offs among major BCI readout modalities are summarized in Table 1; tFUS is considered separately as an intervention modality.

**Table 1.** Representative characteristics of major BCI readout modalities

Modality	Temporal characteristic	Spatial characteristic	Access or depth constraint
EEG	Millisecond-scale neural signals; task-dependent online windows [3]	Model-dependent cortical source localization [3]	Intact skull; portable [1,3]
fNIRS	Hemodynamic response peak at approximately 6 s [29]	Centimeter-scale and array-dependent [29]	Approximately 1.5–2 cm cortical penetration; intact skull; portable [29,30]
MEG/OPM-MEG	Millisecond-scale neural signals [31]	~2–5 mm cortical source localization under favorable conditions [31]	Intact skull; magnetically shielded environment [31]
fUS	Hemodynamic readout; 2-Hz online imaging with a 1.5-s decision window [16]	~100×100 μm in-plane resolution; 12.8 × 16 mm field of view [16]	Skull-dependent; the cited primate and human task studies used cranial access [8,9,16]

Recent work on fUS-based movement-intention decoding, closed-loop ultrasonic BCI control, tFUS-enhanced BCI performance in selected tasks, and seizure-oriented acoustic closed-loop intervention suggests that ultrasound can contribute to interface loops beyond isolated imaging or open-loop stimulation [7,13,16,17]. At present, a more realistic near-term translational route is a hybrid role: EEG can provide fast state tracking, fNIRS can support wearable hemodynamic monitoring, and ultrasound can contribute spatially localized readout, calibration, focal modulation, or event-driven intervention. Accordingly, ultrasound BCI should be evaluated less by single-session decoding accuracy or isolated stimulation effects than by system-level criteria, including reproducible transcranial targeting, stable acoustic coupling, traceable acoustic exposure, measurable modulation responses and closed-loop control rules aligned with task or therapeutic objectives [15,18,19].

## Evidence Maturity and System Gaps

Studies over the past decade show that ultrasound can contribute to the readout, write-in, and closed-loop intervention roles of brain interfaces, but the evidence remains uneven in maturity. fUS readout has advanced from functional brain mapping to single-trial movement-intention decoding and closed-loop ultrasonic BCI, whereas tFUS write-in has shown human cortical modulation, BCI task enhancement in selected settings, and emerging human deep-brain neuromodulation systems [5–7,11–14,16]. Disease-oriented acoustic intervention further suggests that ultrasound stimulation can enter therapeutic feedback loops in early demonstrations [17]. These studies differ substantially in invasiveness, loop structure, deployment condition, and clinical readiness; translational value should therefore be judged by targeting reproducibility, coupling stability, dose traceability, response reliability, and long-term safety monitoring.

Table 2 groups ultrasound-BCI-related systems into 5 functional roles: fUS readout, tFUS write-in, ultrasound-enhanced BCI, closed-loop acoustic intervention, and bidirectional ultrasound BCI. This classification emphasizes that ultrasound BCI should be defined by system function rather than by a single device, imaging mode, or stimulation method.

**Table 2.** Evidence maturity and translational gaps of ultrasound BCI

Domain	Interface role	Most mature evidence	Main translational gap
fUS readout	Brain-state sensing and functional readout	fUS brain mapping; awake-primate task imaging; single-trial movement-intention decoding; closed-loop ultrasonic BCI [5–7,16].	Hemodynamic delay; motion artifacts; coupling drift; cross-day stability; cranial-window and mobile human fUS remain feasibility demonstrations [8,9].
tFUS write-in	Focal acoustic neuromodulation	Human cortical modulation; primate deep-brain modulation; V5-tFUS BCI enhancement; human deep-brain systems [11–14].	Mechanisms and parameter dependence; sensory confounds; off-target effects; skull-dependent field variability; dose reproducibility [4,15,18,32].
Ultrasound-enhanced BCI	Modulation-assisted BCI	Improved visual-motion BCI performance in a controlled human tFUS study [13].	Task specificity; validation of feature-based attention as a candidate mechanism; control of sensory cues, expectancy and training effects [13,32].
Closed-loop acoustic intervention	State-triggered therapeutic intervention	Closed-loop ultrasonic BCI and seizure-oriented acoustic intervention as early interface-loop evidence [16,17].	State–action–response standards; loop latency; trigger rules; exposure reporting; long-term safety monitoring [16–18,28].
Bidirectional ultrasound BCI	Integrated read–write closed-loop control	Conceptual basis from fUS readout, tFUS write-in, acoustic planning, and response monitoring [7,9,10,12–16].	No deployable human system yet integrates localization, dose-constrained write-in, real-time assessment, and adaptive closed-loop control [8–10,13,14].

Closed-loop control may add value over fixed open-loop stimulation when a reliable brain-state biomarker can guide stimulation timing or parameters, improving response or limiting unnecessary exposure. Precedents include adaptive deep brain stimulation (DBS) benefit over optimized continuous DBS, state-dependent TMS plasticity, and responsive neurostimulation efficacy versus sham, although only the DBS study directly compared adaptive and continuous stimulation [20–22]. These findings motivate, but do not establish, benefit for ultrasound, which should be tested against optimized open-loop controls.

## Future Directions of Ultrasound BCI

**Fig. 1.**
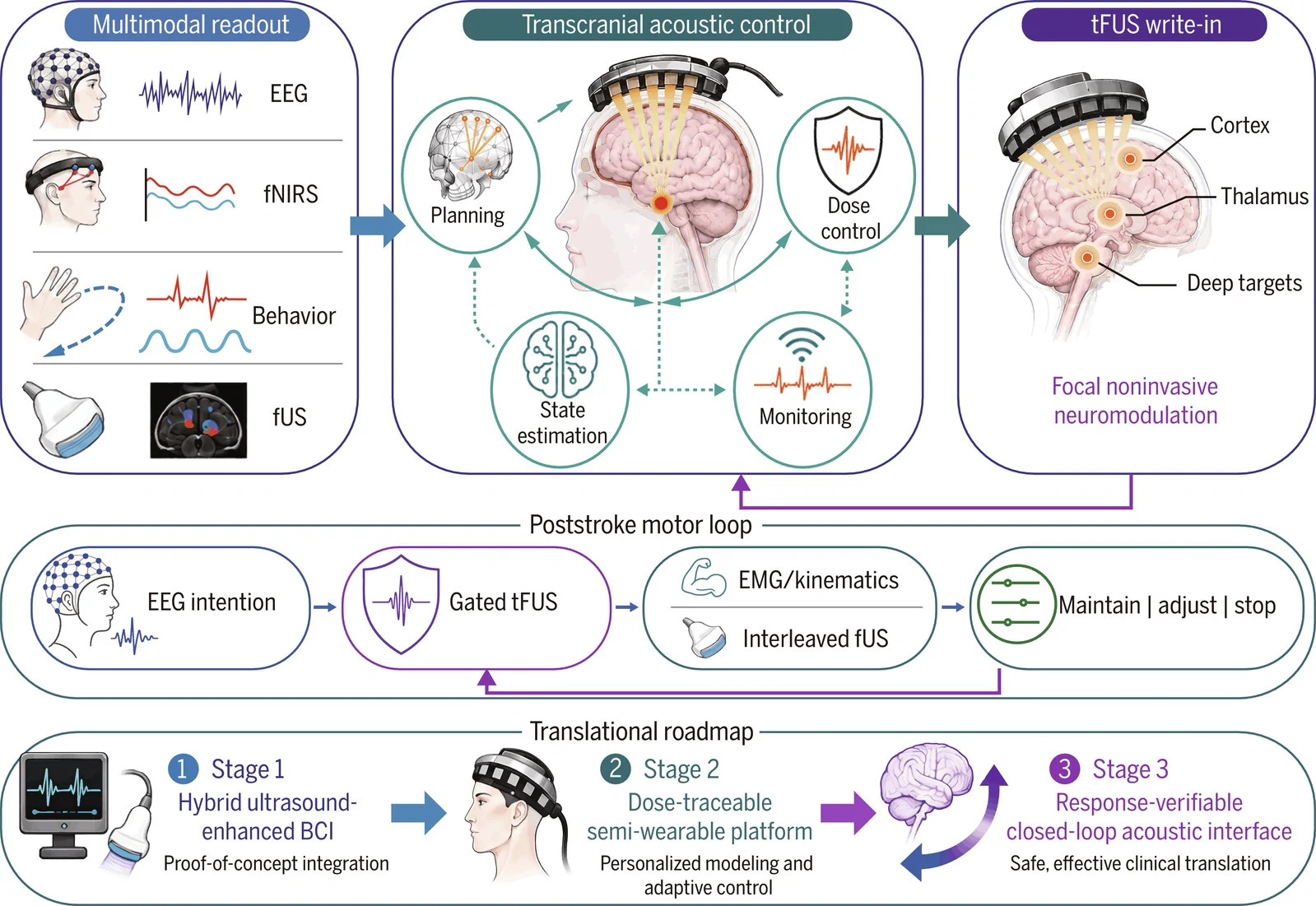
Proposed ultrasound-BCI system architecture, illustrative poststroke motor loop, and translational roadmap. The motor loop is conceptual and has not been clinically validated.

The next stage of ultrasound BCI should be defined by a small number of verifiable engineering tasks rather than by modality-driven enthusiasm. Current evidence shows that ultrasound can enter brain interfaces, but not that ultrasound BCI has matured into a deployable platform. Future development should therefore focus on 3 questions: how ultrasound can augment existing BCIs, which testbeds best reveal its translational value, and how to build platforms with verifiable acoustic fields, traceable acoustic exposure, and measurable closed-loop responses. Fig. 1 integrates the proposed system architecture, an illustrative poststroke motor loop, and a staged translational roadmap.

### From replacement to hybrid ultrasound-augmented BCI

The most realistic near-term form of ultrasound BCI is a hybrid, ultrasound-augmented interface rather than a purely ultrasonic interface. EEG can provide fast online readout, fNIRS can support wearable hemodynamic monitoring, and behavioral or physiological signals can supplement task and system-state information. Ultrasound can then contribute spatially localized readout or acoustic intervention, supported by individualized calibration and planning. Communication and spelling tasks provide established benchmarks for online accuracy and throughput, end-to-end latency, cross-session reliability, user burden, and the incremental benefit of adding ultrasound. However, because these tasks primarily require rapid, portable neural readout already well served by EEG, they are better suited to evaluating ultrasound augmentation than to serving as near-term replacement targets [1,3]. The value of ultrasound is not to continuously increase command throughput, but to add deep-target accessibility, focal adjustability, measurable response, and individualized optimization to existing BCIs. This hybrid division of sensing, estimation, and acoustic intervention is reflected in the system architecture in Fig. 1.

Beyond classification, artificial intelligence (AI) could support individualized acoustic-field planning, multimodal state estimation, patient-specific response prediction, and safety-constrained control. Direct validation in ultrasound BCI remains limited: TUSNet predicts 2-dimensional transcranial pressure fields and phase corrections from numerical data, whereas a reinforcement-learning DBS controller was evaluated only in a computational basal ganglia–thalamic model [23,24]. We therefore propose that future learned controllers integrate anatomy, acoustic propagation, neurovascular responses, and task performance while operating within prespecified dose and safety limits [18].

### From broad possibilities to high-value testbeds

Motor rehabilitation and intervention for abnormal brain states are prioritized as validation testbeds because they combine unmet clinical need with measurable state and response biomarkers, compatible control timescales, and feasibility of repeated closed-loop evaluation. Motor rehabilitation provides repeatable training trials and quantifiable motor responses, whereas abnormal brain states provide event-defined biomarkers suitable for state-triggered intervention. This prioritization reflects their suitability for validation rather than established clinical maturity. Poststroke motor rehabilitation is an established BCI context whose slower adaptation timescale can accommodate the hemodynamic delay of fUS [25]. In one illustrative loop, EEG-detected motor intention would, after signal and safety gates are satisfied, trigger tFUS delivery using a prevalidated setting; electromyography (EMG) or kinematics would quantify the trial response, while interleaved fUS without concurrent tFUS would provide localized posttrial or block-level hemodynamic assessment. At block or session boundaries, prespecified motor-response, fUS-response, and safety thresholds would determine whether to maintain the setting, permit a one-step adjustment within the prevalidated parameter set, or stop stimulation; clinical outcomes would guide longitudinal recovery-based updating (Fig. 1). tFUS could target the motor cortex, thalamus, cerebellum, or related network nodes [4,12,14]; however, this architecture remains unvalidated, and comparable fUS performance through the intact human skull remains unproven [10,16].

Closed-loop intervention for abnormal brain states, such as epilepsy, provides a second high-value testbed because event-defined EEG biomarkers can support state-triggered intervention, as illustrated in a recent preclinical acoustic BCI study [17]. Here, the goal is safe, focal, and monitorable intervention rather than continuous high-speed command output, with clearly defined biomarkers, trigger rules, acoustic-exposure boundaries, and therapeutic endpoints. Neuroergonomics, attention modulation, consciousness assessment, and deep neurofeedback remain relevant extensions, but currently have less direct translational evidence than motor rehabilitation and abnormal-state closed-loop intervention.

### From acoustic-exposure reporting to dose-traceable closed-loop control

Transcranial acoustic-field constraints are prerequisites for ultrasound BCI platforms, not secondary technical details. The adult skull attenuates acoustic energy, distorts the wavefront, shifts the focus, and introduces substantial interindividual variability, thereby affecting both fUS readout and tFUS write-in [15]. Stable transducer fixation, transducer engineering, and acoustic coupling are likewise essential for reproducible use across sessions [19,26]. Wearable ultrasound development highlights important clinical, safety, and engineering constraints [19], whereas emerging wearable-ultrasound concepts suggest that semi-wearable or bedside systems may be an important transitional form before fully mobile head-worn deployment [27]. As outlined in the translational roadmap in Fig. 1, development may progress from hybrid ultrasound-enhanced BCI toward dose-traceable semi-wearable systems and ultimately response-verifiable closed-loop platforms.

Dosimetry and safety monitoring must become integral parts of the interface rather than supplementary information reported after experiments. Because ultrasound neuromodulation is sensitive to acoustic pressure, pulse parameters, beam geometry, skull propagation, and target location, safety frameworks should rely on standardized parameter reporting, thermal and mechanical risk assessment, and longitudinal data accumulation [18]. Following the International Transcranial Ultrasonic Stimulation Safety and Standards Consortium (ITRUSST) reporting recommendations [28], studies should report the transducer and drive configuration, free-field pressure and pulse timing, and individualized estimates of in situ pressure and intensity. Skull effects should be quantified by reporting insertion loss, focal shift and focal geometry, off-target exposure, and model uncertainty. For closed-loop operation, each time-stamped controller action should be linked to the corresponding skull-adjusted field estimate, transducer registration and coupling status, and safety state. Only when these elements are jointly established can ultrasound BCI move from separate demonstrations of imaging or neuromodulation toward a dose-controlled and response-verifiable bidirectional acoustic neuromodulation platform.

## Summary

Ultrasound BCI should be positioned as a bounded engineering route toward safe, effective, and closed-loop transcranial acoustic neuromodulation, rather than as an all-ultrasound replacement for established BCI technologies. Existing evidence supports the feasibility of fUS readout, tFUS write-in, and acoustic closed-loop intervention, but translation will depend on task-specific benefit, reproducible transcranial targeting, stable coupling, dose traceability, closed-loop control rules, and long-term safety monitoring.

Near-term value is most likely to emerge in tasks that require deep-target accessibility, focal modulation, image-guided localization, intra-procedural feedback, and dose constraints, rather than in mature EEG-dominant scenarios such as high-speed spelling or low-cost portable control. Motor rehabilitation and closed-loop intervention for abnormal brain states should therefore serve as priority testbeds, with consciousness assessment, deep neurofeedback, and long-term neural-state monitoring developed as stepwise extensions.

In the longer term, ultrasound may help shift BCI from readout-centered tools toward transcranially targetable, focally controllable, quantitatively monitorable, and adaptively updated acoustic neuromodulation platforms. Achieving this goal will require the joint maturation of acoustic-field modeling, transducer and coupling engineering, real-time signal processing, AI-assisted control, dosimetry standards, safety monitoring, and wearable or semi-wearable ultrasound integration.
